# Association between Facebook Dependence and Poor Sleep Quality: A Study in a Sample of Undergraduate Students in Peru

**DOI:** 10.1371/journal.pone.0059087

**Published:** 2013-03-12

**Authors:** Isabella Wolniczak, José Alonso Cáceres-DelAguila, Gabriela Palma-Ardiles, Karen J. Arroyo, Rodrigo Solís-Visscher, Stephania Paredes-Yauri, Karina Mego-Aquije, Antonio Bernabe-Ortiz

**Affiliations:** School of Medicine, Faculty of Health Sciences, Universidad Peruana de Ciencias Aplicadas – UPC, Lima, Peru; Charité Universitätsmedizin Berlin, NeuroCure Clinical Research Center, Germany

## Abstract

**Objectives:**

Internet can accelerate information exchange. Social networks are the most accessed especially Facebook. This kind of networks might create dependency with several negative consequences in people’s life. The aim of this study was to assess potential association between Facebook dependence and poor sleep quality.

**Methodology/Principal Findings:**

A cross sectional study was performed enrolling undergraduate students of the Universidad Peruana de Ciencias Aplicadas, Lima, Peru. The Internet Addiction Questionnaire, adapted to the Facebook case, and the Pittsburgh Sleep Quality Index, were used. A global score of 6 or greater was defined as the cutoff to determine poor sleep quality. Generalized linear model were used to determine prevalence ratios (PR) and 95% confidence intervals (95%CI). A total of 418 students were analyzed; of them, 322 (77.0%) were women, with a mean age of 20.1 (SD: 2.5) years. Facebook dependence was found in 8.6% (95% CI: 5.9%–11.3%), whereas poor sleep quality was present in 55.0% (95% CI: 50.2%–59.8%). A significant association between Facebook dependence and poor sleep quality mainly explained by daytime dysfunction was found (PR = 1.31; IC95%: 1.04–1.67) after adjusting for age, sex and years in the faculty.

**Conclusions:**

There is a relationship between Facebook dependence and poor quality of sleep. More than half of students reported poor sleep quality. Strategies to moderate the use of this social network and to improve sleep quality in this population are needed.

## Introduction

Internet is involved in many of the people’s routine activities, by facilitating information access and promoting communication; thus, it has been crucial in changes in social development. The largest proportion of Internet users is young people; for example, in Spain, around 98% of adolescences aged between 11 to 20 years reported using Internet [1].

Social networks have developed very fast with a great impact on young people [2]. Among these websites, we found MySpace, Twitter and Facebook; the latter with the largest number of users. Official statistics show that until December 2012, Facebook had 1 billion monthly active users [3]. Currently, in Peru, there are almost 10 million active users, locating in the 24th place worldwide according to Socialbakers statistics [4].

Facebook has several advantages, based on the free access, facilitating communication as well as sharing personal information. However, the excessive use of this kind of social networks might cause several consequences including misuse, dependence and addiction [5], as well as potentially affecting life and sleep quality. Previous studies have shown that the use of electronic media, such as television, personal computers, Internet, and computer games, is associated with sleep disorders [6], [7], [8]. Mechanisms for this association are diverse and include the use of several hours among dependent people altering sleep patterns [9], the gambling activities that are provided by Facebook platform [5], among others. However, young people are not even aware of the adverse effects of using electronic media [10]. Both the quantity and the quality of sleep might have a strong influence on mood and subjective well-being [11]. Especially, in the case of young people, a poor sleep quality could have an impact on academic performance as well [12].

Some research has suggested the influence of internet misuse on insomnia and other sleep disturbances: increased time spent on the Internet disrupted the sleep-wake schedule [13]. Based on this, we hypothesized that Facebook misuse might alter sleep quality. To our knowledge, no previous studies have been found linking Facebook use and sleep quality. The main objective of this study was to evaluate the association between Facebook dependence and sleep quality among a sample of undergraduate students in a private university. In addition, we determined the prevalence of Facebook dependence and poor quality of sleep in this population.

## Materials and Methods

### Study Design, Setting and Participants

A cross-sectional study was performed involving undergraduate students at the Universidad Peruana de Ciencias Aplicadas, Lima, Peru. Potential participants were those registered in the School of Psychology, part of the School of Health Sciences. A complete census was performed including those who accepted to participate in the study.

### Variables Definition

The outcome of interest was sleep quality, defined as good or poor according to the Pittsburgh Sleep Quality Index (PSQI) [14]. This instrument has been previously validated and adapted to Spanish in Mexico [15], and Colombia [16], with a good reliability score (Cronbach’s alpha = 0.78) and significant component-total score correlations [15]. In addition, this instrument has been previously used in our context to also assess health science students [17]. A total of 21 questions were used to determine the seven components of sleep quality: duration, disturbance, latency, day dysfunction due to sleepiness, sleep efficiency, overall sleep quality and medication use. A global score of 6 or greater was defined as the cutoff to determine poor sleep quality as previously reported [14], [15], [16], [17], [18].

The exposure of interest was Facebook dependence. The questionnaire of Internet addiction, developed in Spanish by Enrique Echeburúa [19], was adapted to the Facebook case for our study purposes. The instrument comprised 8 two-choice questions (yes/no). This questionnaire is mainly focused on worries, concerns, satisfaction, time of use and efforts to reduce it, control, and other activities due to Facebook use; we, therefore, utilized as a measure of Facebook dependence [1]. A cutoff of 5 or more was used to establish the presence of dependence as previously reported [20].

Other variables considered in the analysis were age (in years), sex (male/female), and years in the faculty (from one to six).

### Procedures and Data Collection

A complete census was planned to enroll the appropriate sample size. Students were contacted in their classroom before or after lectures. Previous informed consent, a self-applied questionnaire was provided to participants for information and data collection. This process took around 10 to 15 minutes. After that, a quick review of the questionnaire with the participant was performed to guarantee appropriate completeness.

### Sample Size

Assuming 10% of Facebook misuse prevalence and 60% of poor sleep quality [18], a total of 385 participants were required to find an association of 3 or more with 5% of significance and 80% of power (PASS 2008, NCSS, Utah, US). When a rejection rate of 5% was considered, around 405 participants were needed.

### Statistical Analysis

After data collection, a double data entry process was performed using Microsoft Excel 2010 for Windows, and then data was transferred to STATA 11 (STATA Corp, College Station, TX, US) for analysis. First, a description of the study population was performed using means and proportions to compare characteristics according to sleep quality, our outcome of interest. Second, internal consistency, assessed by Cronbach’s alpha, of the Facebook dependence and the PSQI questionnaires was calculated and reported. In the case of Facebook questionnaire, all the questions were evaluated together; whilst, in the case of the PSQI, only questions with categorical response options, and not numerical (number of hours) were used for calculation. Then, prevalence and 95% confidence intervals (IC95%) of the variables of interest were calculated. Finally, the association between Facebook dependence and poor sleep quality was evaluated using generalized linear models and reporting prevalence ratios (PRs) and 95% confidence intervals (95%CI) adjusted for potential confounders.

### Ethics

This project was reviewed and approved by the Ethical Committee of the Universidad Peruana de Ciencias Aplicadas, Lima, Peru. An oral informed consent was used to explain the purpose of the study. Data was collected without personal identifiers to guarantee appropriate confidentiality.

## Results

A total of 428 participants were enrolled. Of them, 10 questionnaires were excluded because of inconsistencies. Thus, only 418 (97.6%) were analyzed. The mean age of participants assessed was 20.1 (SD: 2.5) years, whereas 322 (77.0%) were women. Details comparing the sociodemographic variables assessed according to quality of sleep are shown in Table 1.

**Table 1:** Characteristics of the study population according to sleep quality (N = 418).

	Sleep Quality		
	Good (n = 188)	Poor (n = 230)	p-value*
Sex (n, %)			
Female	144 (44.7%)	178 (55.3%)	0.85
Male	44 (45.8%)	52 (54.2%)	
Age (years)			
Mean (SD)	20.0 (2.4)	20.1 (2.7)	0.74
Years in the faculty (n, %)			0.70
One/two	78 (42.6)%	105 (57.4%)	
Three/Four	82 (46.9%)	93 (53.1%)	
Five/Six	28 (46.7%)	32 (53.3%)	
Facebook dependency (n, %)*			0.08
No	177 (46.3%)	205 (53.7%)	
Yes	11 (30.6%)	25 (69.4%)	

* Fisher’s exact test was used for p-value calculation.

Internal consistency, assessed by Cronbach’s alpha, was 0.67 for Facebook questionnaire, whereas it was 0.71 for the Pittsburgh Sleep Quality Index. Furthermore, prevalence of Facebook dependence was 8.6% (95%CI: 5.9%–11.3%), while the prevalence of poor sleep quality was 55.0% (95% CI: 50.2%–59.8%).

Table 2 shows the mean scores of each of the components of the Pittsburgh Sleep Quality Index compared by Facebook dependency. Of note, the daytime dysfunction component was the only statistically significant between groups of comparison (p = 0.007). In our multivariable Poisson regression model, after controlling for age, sex and years in the faculty, a significant association between Facebook dependence and poor sleep quality was found, (PR = 1.31, 95% CI 1.04–1.67). See details in Table 3.

**Table 2:** Score of the Pittsburgh Sleep Quality Index components according to Facebook dependency.

	Facebook dependency		
	Yes (n = 36)	No (n = 382)	p-value*
**Component 1:** Subjective quality of sleep			
Mean (± SD)	1.19 (±0.03)	1.31 (±0.10)	0.28
**Component 2:** Sleep latency			
Mean (± SD)	0.97 (±0.15)	1.10 (±0.04)	0.38
**Component 3:** Sleep duration			
Mean (± SD)	0.58 (±0.13)	0.64 (±0.05)	0.73
**Component 4:** Habitual sleep efficiency			
Mean (± SD)	0.63 (±0.18)	0.62 (±0.05)	0.89
**Component 5:** Sleep disturbance			
Mean (± SD)	1.17 (±0.06)	1.13 (±0.02)	0.59
**Component 6:** Use of sleeping medication			
Mean (± SD)	0.25 (±0.10)	0.34 (±0.04)	0.53
**Component 7:** Daytime dysfunction			
Mean (± SD)	1.56 (±0.12)	1.19 (±0.04)	0.007
**Total:** Sum of the components			
Mean (± SD)	6.47 (±0.50)	6.20 (±0.15)	0.60

* T test for independent samples was used for p-value calculation.

**Table 3:** Association between Facebook dependency and poor sleep quality: Crude and adjusted models.

	Crude model	Adjusted model*
	PR (95%CI)	PR (95%CI)
**Facebook dependency**		
No	1 (Reference)	1 (Reference)
Yes	1.29 (1.02–1.64)	1.31 (1.04–1.67)

* Adjusted for age, sex and years in the faculty.

## Discussion

In this study, we have demonstrated a relationship between Facebook dependence and poor quality of sleep. Thus, Facebook dependent participant had about 1.3 times greater prevalence of poor sleep quality than the non-dependent group, after controlling for age, sex and years in the faculty. As a result, the poor sleep quality prevalence of 53.7% among students not classified as Facebook dependent increases to 69.4% among those classified as Facebook dependent (i.e., an absolute increase of 15.7 percentage points). Moreover, most of the potential impact of the Facebook misuse on sleep quality seems to be in the daytime component. Thus, students with Facebook dependence have more daytime dysfunction that those without dependence. To our knowledge, this is the first study reporting association between these two variables. In addition, 8% of students had Facebook dependence and more than half of them had poor sleep quality.

There are several potential explanations regarding the association found in this study. First, addicted Facebook users are likely to use it anywhere from several hours; thus, to accommodate such excessive use, sleep patterns are typically disrupted due to late night logins [9], which can explain the statistically difference found in the daytime function. In this sense, leisure activities that are unstructured, especially in young people, seem to be negatively related to good sleep patterns [6]. Second, some activities in the Facebook website, such as, messaging friends, playing games, and others, can engage person in misuse and addiction [5]. In this case, Facebook itself works as the bridge between gambling activities and sleep disorders. Third, persons with addition to online video games might develop feelings of loneliness and isolation, which has been also associated with sleep fragmentation [21]. Finally, it has been suggested that exposure to bright light at the wrong time of the day can alter circadian rhythm sleep with insomnia and excessive sleepiness [22].

Despite of the potential impact of social networks and their misuse, it is remarkable that little information is available regarding the potential effect of them, especially Facebook, on lives and lifestyles of young people [23]. Our results suggest that about 8% of our sample of undergraduate students might have some degree of Facebook addiction. A previous study found that extroverted individuals reported greater addictive tendencies to Facebook [24]. Moreover, in some cases, the Facebook dependence has been associated with relationship frustration as well as the emergence of jealousy [25]. Disclosure of sensitive information could also have an impact on people performance [26]. However, some literature also suggests that the term Facebook dependence and addiction, similar to the term Internet addiction, is not a correct term because there are several activities that can attract young people to the website [5]. Therefore, specific activities in Facebook such as messaging friends, playing games, gambling, among others, might be involved in the addiction activities rather than a particular website. In general, there is a minimization of the harmful consequences of the Facebook dependence, not only at the health level, such as drinking [27], smoking, substance abuse, sedentary lifestyles, depression [28], suicide [29], and poor academic performance [30], but also, at other levels like privacy reduction, isolation, exposing children to online predators, etc. Therefore, new strategies and other studies are needed to reduce the impact of social networking sites on people.

Our findings also show that more than half of the undergraduate students had poor sleep quality. Other studies have found similar results using this kind of population and the same instrument [17], [18], [31]. For example, a study in Taiwan found a prevalence of 54.7% among incoming undergraduate students [18], and similarly, a study in adolescences aged between 16 and 19 years found 52.9% of prevalence in Sao Paulo, Brazil [31]. A previous study in Peru involving medical students found that poor sleep quality rates and daily somnolence were greater during hospital based practices compared to vacations periods [17]. However, although sleep quality scores improved during holidays, daily somnolence did not. Poor sleep quality might potentially have impact on student learning capacity as well as academic and working performance [12], [32].

This might be the first study finding association between Facebook dependence and poor sleep quality. Strength of this study also includes the use of a well-known scale to assess poor sleep quality as the Pittsburgh Sleep Quality Index. However, this study has several limitations. First, the study design, cross sectional in nature, can only determine association and not causality. Although we used regression models and adjusted for potential confounders, further longitudinal studies are needed to confirm our findings. Second, the scale utilized to assess Facebook dependence was not specific for this. We decided to adapt a questionnaire for Internet addiction validated in Spanish for our purposes. In addition, questions regarding this scale were applying focused only on Facebook use and not other social networks or gaming sites. Recently, a scale for Facebook addiction has been published [33]; however, it has not been validated in Spanish restricting its use for this study. Third, although Spanish versions of the scales were used and, the Pittsburgh scale has been previously used in our country, scales used to assess Facebook dependency and sleep quality were not validated in Peru. Finally, although our models were adjusted by some potential confounders (age, sex, and years in the faculty), other variables associated with social networks dependence and sleep quality, such as level of education [31], social support, and socioeconomic status [34] have not been considered. In the case of socioeconomic status, students belong to the upper socioeconomic quintile in Lima. So, the effect of this variable might be minimal.

In conclusion, there is an association between Facebook dependence and poor quality of sleep. Additionally, approximately one in 10 students might have addiction to Facebook, while over 55% had poor sleep quality. We suggest further studies to corroborate these findings and develop strategies to moderate the use of this social network and to improve sleep quality in this population.
